# Unusual presentation of medication‐induced atrial fibrillation: A case report

**DOI:** 10.1002/ccr3.9204

**Published:** 2025-01-26

**Authors:** Thamir Hashim, Yavuz Yigit, Khalid Yasir Fadul, Mostafa Dayraki, Ashraf Elmalik, Baha Hamdi Alkahlout, Aftab Mohammad

**Affiliations:** ^1^ Department of Emergency Medicine Hamad Medical Corporation Doha Qatar; ^2^ Blizard Institute Queen Mary University London United Kingdom; ^3^ School of pharmacy University of Colorado Boulder Colorado USA; ^4^ College of Medicine Qatar University Doha Qatar

**Keywords:** alpha‐1 adrenergic antagonists, emergency medicine, medication‐induced atrial fibrillation, nephrolithiasis complications, tamsulosin adverse effects

## Abstract

This case highlights that atrial fibrillation can occur as an adverse effect of tamsulosin even in younger patients at lower doses, challenging the conventional understanding that this complication primarily affects older individuals on higher doses. Clinicians should remain vigilant for this potential side effect across all patient demographics to ensure prompt identification and management.

## 1 INTRODUCTION

Medication‐induced atrial fibrillation is a recognized but rare adverse effect of alpha‐1 adrenergic antagonists, predominantly associated with elderly patients and higher doses. We present a case of atrial fibrillation induced by a 0.4 mg dose of tamsulosin in a 33‐year‐old male with no previous history of arrhythmias. The patient was prescribed tamsulosin for nephrolithiasis, and shortly after taking the medication, he experienced atrial fibrillation, which spontaneously reverted to sinus rhythm without medical intervention.

This case challenges the conventional belief that medication‐induced atrial fibrillation primarily occurs in older patients at higher doses. It highlights the importance of considering this adverse effect in a broader patient demographic and underscores the significance of promptly identifying the causative medication in emergency settings.

## 2 CASE HISTORY

**FIGURE 1:**
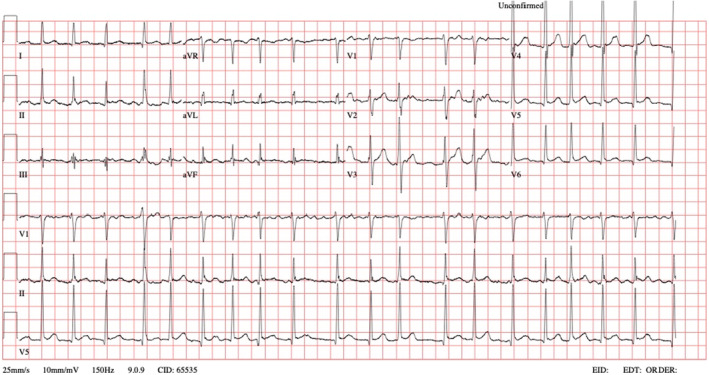
The initial ECG displays atrial fibrillation.

A 33‐year‐old male with an unremarkable past medical history, except for nephrolithiasis, was brought to the Emergency Department (ED) in the morning by Emergency Medical Services (EMS). He complained of palpitations that began around midnight the same day. An Electrocardiogram (ECG) performed by EMS revealed atrial fibrillation with a heart rate ranging from 113 to 149 beats per minute (BPM). Upon assessment, the patient had an irregular peripheral pulse rate of 118 BPM, a blood pressure of 105/72 mmHg, and a peripheral oxygen saturation of 100%. He was alert and oriented, with a Glasgow Coma Scale score of 15, and did not report chest pain, dyspnea, or any other symptoms. A subsequent ECG confirmed atrial fibrillation with a rapid ventricular response (Figure 1). The patient denied any risk factors for atrial fibrillation, such as gastroesophageal reflux, alcohol abuse, and there were no signs of infection.

### 2.1 Methods

Upon further evaluation, the patient disclosed that he had been prescribed Tamsulosin 0.4 mg once daily as part of the treatment regimen for his renal colic and nephrolithiasis. Last night, he ingested two tablets of the medication. With a body mass index of 26, he had been on this medication for the past 10 days. Since initiating this treatment, he reports the absence of pain associated with renal colic.

**FIGURE 2:**
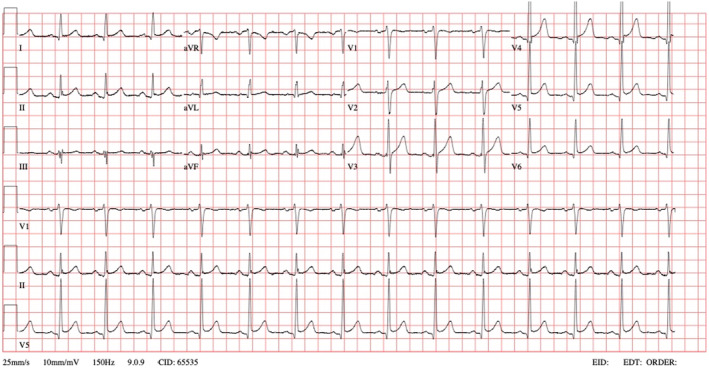
Atrial fibrillation spontaneously reverted to sinus rhythm 30 min after the initial ECG, with no medication administered during this time.

The patient subsequently reverted to normal sinus rhythm (Figure 2) without any medical intervention within a 30‐min period, approximately 10 h after the onset of palpitations. Upon reassessment, his condition was found to be normal, and laboratory results, including a complete blood count, renal and liver function tests, and serum sodium, potassium, chloride, and thyroid function tests, were unremarkable. He was discharged home with a recommendation for cardiology follow‐up for further evaluation. The patient was advised to discontinue Tamsulosin until further investigations are conducted.

He was followed up by cardiology where transthoracic echocardiogram was unremarkable for any structural disease, and holter monitor was uneventful.

### 2.2 Conclusion and results

Our case illustrates an intriguing deviation from the typical profile of medication‐induced atrial fibrillation. By highlighting the risk associated with a lower dose and younger age, it contributes to the ongoing discussion on the adverse effects of alpha‐1 adrenergic antagonists in a broader patient demographic.

## 3 DISCUSSION

Atrial fibrillation stands as the predominant cardiac arrhythmia globally, contributing significantly to both morbidity and mortality.^1^, ^2^, ^3^ The Emergency Department (ED) is a hectic place and early identification of these patients in the emergency department (ED) is vital.^4^, ^5^ The most common precipitants of atrial fibrillation include surgery and pneumonia. Rarer causes include medication side effects, cardiovascular disease, respiratory illness, metabolic disorders, and illicit drug use.^6^

Alpha1 adrenoceptors blockers like tamsulosin and alfuzosin are commonly prescribed as a first‐line agent for symptomatic benign prostatic hypertrophy (BPH), a condition that affects an estimated three out of every four men greater than 70 years old.^7^ While it is generally well tolerated with limited serious side effects, post‐marketing reports have identified atrial fibrillation as a rare but potentially life‐threatening adverse drug related (ADR) effect of medication. Further, in a summary of adverse event data on clinical pharmaceutical trials of tamsulosin, atrial fibrillation was reported to occur in 0.1%–0.6% of patients.^8^ This ADR is expected with elderly who have multiple medications with possible interaction.

Atrial fibrillation often manifests following acute precipitants such as surgery, sepsis, or pneumonia. While medication‐induced atrial fibrillation is less common, it does exist and merits careful consideration. Alpha‐1 adrenergic antagonists, including tamsulosin, function by blocking alpha‐1 receptors, thereby lowering blood pressure, making them valuable for the management of BPH and expelling ureteral stones. Our case, however, introduces an intriguing dimension.

Domingos et al.^9^ demonstrated a potential link between autonomic dysfunction and idiopathic recurrent kidney stone formers, which theoretically could predispose individuals to atrial fibrillation. In the case of our patient, he experienced nephrolithiasis for the first time and did not report any pain in the preceding week that could have resulted in a sympathetic system activation.

Clinical trials and post‐marketing surveillance have linked tamsulosin, an alpha‐1 adrenergic antagonist, to side effects such as dizziness, headache, orthostatic hypotension, palpitations, and reflex tachyarrhythmias.^10^ Notably, our patient, a 33‐year‐old male with no prior history of arrhythmias, was prescribed tamsulosin at a relatively low dose (0.4 mg once daily) for nephrolithiasis. This contrasts with the case reported by McGuire et al.,^8^ which described a 61‐year‐old patient developing atrial fibrillation attributed to tamsulosin. Our case underscores the possibility of this adverse effect occurring in a younger patient population.

In an emergency medicine setting, identifying the instigating medication, as in the case of tamsulosin, can not only inform acute management but also guide the long‐term evaluation and treatment. Patients, especially those atypical for this adverse effect, should be educated about the potential risks, enabling them to monitor their heart rate and blood pressure at home, they should be encouraged to maintain a dialogue with their prescribing physicians, allowing for informed decision‐making and a proactive approach to their healthcare.

## AUTHOR CONTRIBUTIONS

**Thamir Hashim:** Data curation; methodology; supervision. **Yavuz Yigit:** Methodology; supervision; writing – original draft; writing – review and editing. **Khalid Yasir Fadul:** Data curation; formal analysis; methodology. **Mostafa Dayraki:** Data curation; formal analysis; investigation. **Ashraf Elmalik:** Writing – original draft. **Baha Hamdi Alkahlout:** Conceptualization; methodology; writing – review and editing. **Aftab Mohammad:** Methodology; writing – original draft; writing – review and editing.

## FUNDING INFORMATION

The authors declare that they have no known competing financial interests or personal relationships that could have appeared to influence the work reported in this paper.

## CONFLICT OF INTEREST STATEMENT

The authors declare no competing interests.

## CONSENT

Written informed consent was obtained from the patient to publish this report in accordance with the journal's patient consent policy.

## Data Availability

The data that support the findings of this study are available from the corresponding author upon reasonable request.
